# Occurrence and Characterization of Small Microplastics (<100 μm), Additives, and Plasticizers in Larvae of Simuliidae

**DOI:** 10.3390/toxics10070383

**Published:** 2022-07-10

**Authors:** Fabiana Corami, Beatrice Rosso, Valentina Iannilli, Simone Ciadamidaro, Barbara Bravo, Carlo Barbante

**Affiliations:** 1 Institute of Polar Sciences, CNR-ISP, Campus Scientifico—Ca’ Foscari University of Venice, Via Torino 155, 30172 Venice, Italy; beatrice.rosso@unive.it (B.R.); barbante@unive.it (C.B.); 2 Department of Environmental Sciences, Informatics and Statistics, Ca’ Foscari University of Venice, Via Torino 155, 30172 Venice, Italy; 3 ENEA, SSPT-PROTER-BES, C. R. Casaccia, Via Anguillarese 301, 00123 Roma, Italy; valentina.iannilli@enea.it; 4 ENEA, SSPT-PROTER-BES, C. R. Saluggia, Via Crescentino Snc., 13040 Saluggia, Italy; simone.ciadamidaro@enea.it; 5 ThermoFisher Scientific, Str. Rivoltana, Km 4, 20090 Rodano, Italy; barbara.bravo@thermofisher.com

**Keywords:** blackfly larvae, freshwaters, Simuliidae, microplastics, additives, plasticizers

## Abstract

This study is the first to investigate the ingestion of microplastics (MPs), plasticizers, additives, and particles of micro-litter < 100 μm by larvae of Simuliidae (Diptera) in rivers. Blackflies belong to a small cosmopolitan insect family whose larvae are present alongside river courses, often with a torrential regime, up to their mouths. Specimens of two species of blackfly larvae, *Simulium equinum* and *Simulium ornatum*, were collected in two rivers in Central Italy, the Mignone and the Treja. Small microplastics (SMPs, <100 μm), plasticizers, additives, and other micro-litter components, e.g., natural and non-plastic synthetic fibers (APFs) ingested by blackfly larvae were, for the first time, quantified and concurrently identified via MicroFTIR. The pretreatment allowed for simultaneous extraction of the ingested SMPs and APFs. Strong acids or strong oxidizing reagents and the application of temperatures well above the glass transition temperature of polyamide 6 and 6.6 (55–60 °C) were not employed to avoid further denaturation/degradation of polymers and underestimating the quantification. Reagent and procedural blanks did not show any SMPs or APFs. The method’s yield was >90%. Differences in the abundances of the SMPs and APFs ingested by the two species under exam were statistically significant. Additives and plasticizers can be specific to a particular polymer; thus, these compounds can be proxies for the presence of plastic polymers in the environment.

## 1. Introduction

The ingestion of ubiquitous and persistent microplastics (MPs) in biota, i.e., in macroinvertebrates, is documented in polar environments [1,2,3], marine environments [4,5,6,7,8], and riverine environments [9,10,11,12]. Invertebrates ingest food particles according to the size of their mouthparts; the size of these particles is usually <100 μm. MPs < 100 μm (small microplastics, SMPs), as well as additives, plasticizers, and other micro-litter components <100 μm (e.g., natural and non-plastic synthetic fibers; APFs), can be mistaken for food particles, ingested, and enter the trophic web. SMPs can be primary, e.g., those released from the discharge of washing machines [13], or secondary, e.g., those derived from the fragmentation of macroplastics and large microplastic pieces. It should be underlined that the fragmentation of large MPs can release or expose additives and plasticizers employed in the plastic industry and can be polymer-use specific; these compounds are thought to be responsible for the toxicity of plastic polymers toward biota [14]. However, assessment of the additives and plasticizers in environmental matrices and biota has been overlooked. Some studies have tested the ingestion of MPs by macroinvertebrates in lab conditions, but these controlled exposure studies may lack environmental realism, and the concentration of the ingested MPs cannot correspond to those observed in nature [10]. Hence, the focus of this study is to investigate the ingestion of SMPs and APFs in two blackflies species, *Simulium equinum* (Linnaeus, 1758) and *Simulium ornatum* (Meigen, 1818 (complex)), for the first time. Specimens of these two species were collected in their habitat. 

Blackflies (Diptera, Simuliidae) form a relatively small and uniform family of insects, numbering nearly 2300 known species worldwide [15]. They are passive filter feeders, filtering suspended particulate matter from the water and staying fixed to smooth surfaces in the lotic reaches of watercourses. Blackfly larvae are crucial in watercourses’ ecologies, making the filtered matter available for other invertebrates, amphibians, and fishes that feed on them [16]. Blackfly larvae spend most of their time attached to the substrate in watercourses, and, in this sedentary mode, they feed. The primary feeding device, and distinguishing feature of the family, is a pair of large cephalic filtering “fans,” which are complex oral structures consisting of many serially arranged rays fixed on the two fans’ stems. These filtering “fans” are chitinous–mucous structures. Opened in riverine waters, they can trap fine suspended organic (e.g., detritus, bacteria, algae, animal matter) and inorganic matter with a passive and undiscriminating collection system; if it can be manipulated in the mouth and can enter the *cibarium*, any catchable particulate filtrate is taken into the gut. If compressible, even larger particles can be swallowed [17,18]. Concerning their feeding mode, blackfly larvae may ingest SMPs and APFs.

A previously developed pretreatment method (at CNR-ISP, [7]) was optimized to assess the abundance of SMPs, APFs, and other microlitter components ingested by blackfly larvae; the method allows for concurrent extraction of all the aforementioned particles and does not contribute to these particles’ further degradation/denaturation. Many pretreatment methods employ strong oxidizing agents or strong acids, which can modify particle sizes and contribute to discoloration, degradation, and loss of several polymers [19], especially nylon 6 and nylon 6,6 (PA 6 and PA 6,6). Moreover, these pretreatment methods employ temperatures ≥ 60 °C, which can contribute to the loss of polymers, in particular, PA 6 and PA 6,6, as the range of their glass transition temperature (Tg) is 55–60 °C [7,19,20]. Hence, these pretreatment methods can result in underestimation of the actual abundance of MPs/SMPs in the samples and samples that are not representative. SMPs and APFs will be simultaneously quantified (microscopic count) and identified via Micro-FTIR.

## 2. Material and Methods

### 2.1. Sampling Sites and Macroinvertebrate Sampling

**Figure 1:**
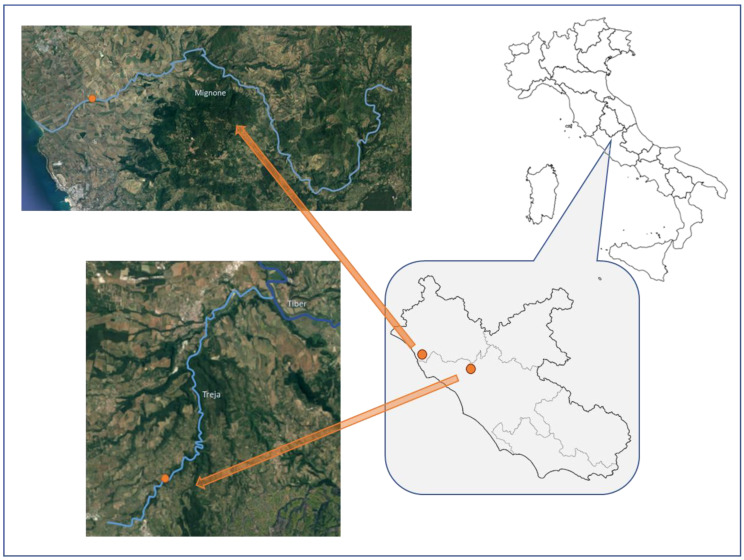
Sampling sites where blackfly larvae (Simuliidae) were collected; the Mignone and Treja rivers are located near Rome, in Lazio, Italy.

When sampling macroinvertebrates for water quality monitoring, organisms of *Simulium equinum* and *Simulium ornatum* were collected in the summer of 2018 from theTreja River (42.18402, 12.37895), a few kilometers downstream from Mazzano Romano, near the Monte Gelato waterfalls, an attractive place for tourists during spring and summer, and the Mignone River (42.19557, 11.79347), near Tarquinia (Figure 1). Because of their characteristics, these rivers may well represent environments influenced by various pressures and impacts.

The Treja River is the third major right tributary of the Tiber River. Its source is in Monte Lagusiello near Lake Bracciano, and the river flows through a valley that gives it its name, which is characterized by tuffaceous material. Along the river’s course, the natural environments are in a good state of conservation; there are alternating areas of cultivated countryside, livestock activities, and woods.

The Mignone River is 62 km long, originating in the Sabatini Mountains in the territory of the town of Vejano, located northwest of Lake Bracciano. In its initial part, this river is almost a stream, which has carved its bed within deep valleys, while the remaining stretch was once navigable. It reaches the Tyrrhenian Sea, north of Rome, in Tarquinia, after a course of 60 km. The river and its catchment area represent one of the most remarkable environmental areas of Lazio, due to high conservational preservation as Sites of Community Importance. However, the qualitative state of the river in the lower course is influenced by anthropogenic activities.

Moreover, they are frequently visited nature reserves, and the entire catchment areas of the Mignone and Treja are the object of historical and artistic tourism. Therefore, agriculture, WWTP (wastewater treatment plant) discharges, and various tourist activities in these two areas may be significant sources of SMPs and APFs.

At each of the sites, which are, as a general rule, monitored for water quality status, macroinvertebrates, including blackfly larvae, were collected using a hand net by placing it on the riverbed and moving the substratum in front of the net opening with the free hand or a foot. Sampling was performed in riffle mesohabitat, which is the most suitable for blackfly larvae according to their ecology [18]. In order to cover the highest diversity of the local habitat conditions where macroinvertebrates and different blackfly species could be found, all microhabitats were surveyed in the riffle mesohabitat, giving priority to the stable substratum on which blackfly larvae can anchor themselves. The finalized sample for each site was sorted in the field to separate the substratum from organisms. All blackfly larvae were sorted among the macroinvertebrates collected; they were immediately fixed in ethanol 70% (absolute, for HPLC, ≥99.8%, Sigma Aldrich, Merck Darmstadt, Germany) to prevent gut content excretion. Different species of blackfly larvae were identified through microscopic morphological examination at the ENEA laboratory. The two species, *Simulium equinum* (Linnaeus, 1758) and *Simulium ornatum* (Meigen, 1818 (complex)), were identified at both sampling sites.

Thirty organisms were collected for each of the two identified species of Simuliidae at each sampling site in the rivers under study. Before their identification, the organisms were carefully rinsed several times with ultrapure water (Milli-Q^®^, Merck Darmstadt, Germany), followed by a fresh 70% ethanol solution to remove materials on the body surface, which were, therefore, not ingested. Then, 10 organisms per species were employed for taxonomic identification and dry weight detection. The average dry weight per organism of *S. ornatum* was 0.5 mg, while for the *S. equinum*, it was 0.6 mg.

The organisms designated explicitly for the analysis of microplastics and other microlitter components (20 organisms per species at each sampling site, which is monitored for water quality) were preserved in ethanol 80% and then transferred to the laboratory of CNR-ISP (spring 2020).

### 2.2. Quality Assurance and Quality Control (QA/QC)

Decontamination and pretreatment procedures were performed at CNR-ISP Venezia in a plastic-free cleanroom ISO 7. This cleanroom (a controlled-atmosphere laboratory where atmospheric pressure, humidity, temperature, and particle pollution are controlled) is entirely free of plastic materials, even in the air pre-filters. The environmental contamination in the pretreatment procedures for the analysis of SMPs and APFs is efficiently minimized.

Samples were pre-treated (extraction and purification) and filtered in batches on aluminum oxide filters (ANODISC filters, Supported Anopore Inorganic Membrane, 0.2 μ, 47 mm, Whatman™; Merck, Darmstadt, Germany). The pretreatment procedures and filtration were performed under a decontaminated steel fume hood. Operators wore cotton lab coats and nitrile gloves. All glassware was previously washed with a 1% Citranox^®^ solution (Citranox^®^ acid detergent, Sigma Aldrich purchased from Merck Darmstadt, Germany), rinsed with ultrapure water (UW, produced by UW system, Elga Lab Water, Veolia, High Wycombe, UK), and decontaminated with acetone (suitable for HPLC, 99.9%, Sigma Aldrich, Merck Darmstadt, Germany). Then, the glassware was rinsed with a 50% (*v*/*v*) solution of methanol (suitable for HPLC, 99.9%, Sigma Aldrich, Merck Darmstadt, Germany) and ethanol (absolute, for HPLC, ≥99.8%, Sigma Aldrich, Merck Darmstadt, Germany), and, finally, with ethanol. The steelware was previously rinsed with UW, decontaminated with methanol, a 50% (*v*/*v*) solution of methanol and ethanol, and ethanol. Reagent (e.g., UW from Milli-Q^®^ (Millipore, Merck, Darmstadt, Germany), ethanol, H_2_O_2_, etc.) and procedural blanks were performed for each batch.

After filtration, all filters were stored in decontaminated glass Petri dishes covered with aluminum foil. Before the analysis, filters were transferred from the fume hood in the cleanroom to the Micro-FTIR laboratory, carefully covered with aluminum foil to avoid any external contamination.

Certified reference materials for MPs in biota are lacking; therefore, to estimate the yield of the pretreatment procedure used in this study, a model organism that was accessible and easy to sample was chosen. The choice was *Monocorophium insidiosum* (Corophidae, Amphipoda), whose specimens were sampled in the Pordelio Channel, Venice Lagoon, in the summer of 2020; three pooled samples were then spiked with silver–grey beads of polyamide 12 (average size 90 μm; Goodfellow Cambridge Limited, Huntingdon, UK). The polymer to be employed was selected by the particle color, size, and ease of mixing it in the sample.

### 2.3. Extraction, Purification, and Filtration of APFs and SMPs Ingested by Blackfly Larvae

For the extraction and purification of the APFs and SMPs ingested by blackfly larvae, the method developed by Corami et al. [7] was employed with slight modifications. Due to the small size of blackfly larvae, the organisms were not dissected; hence, the APFs and SMPs were extracted from the whole organism.

Briefly, under a decontaminated fume hood in the cleanroom, organisms were put in a decontaminated Erlenmeyer flask with H_2_O_2_, ethanol, and UW (1:2:1 ratio) and stirred for 96 h on a multipurpose orbital shaker at room temperature. The aim of this step is not thorough digestion (i.e., strong acids or strong oxidants); rather, it is an extraction of the ingested particles by dissolving the organic matter with no further denaturation of polymers. The residual dissolved organic matter was removed through the following purification procedure: flushing ethanol and a 70% (*v*/*v*) ethanol–methanol solution alternated with the extracted slurry directly onto the aluminum oxide filter during vacuum filtration.

Filtration was performed with a decontaminated glass filtering apparatus and a vacuum pump Laboport^®^ (VWR International, Milan, Italy) under a decontaminated fume hood in the cleanroom; aluminum oxide filters were rinsed by alternating 50 mL of a 50% (*v*/*v*) solution of ethanol with 50 mL of 70% (*v*/*v*) solution of ethanol–methanol before the filtration. The filter was rinsed several times with a 50% (*v*/*v*) ethanol solution at the end of filtration. Each filter was stored in decontaminated glass Petri dishes for at least 72 h under a fume hood in the cleanroom before the analysis via Micro-FTIR.

### 2.4. Quantitative and Chemical Characterization of APFs and SMPs via Micro-FTIR

A Nicolet™ iN™ 10 infrared microscope (Thermo Fisher Scientific, Madison, WI, USA), equipped with an ultra-fast motorized stage and liquid-nitrogen-cooled MCT detector (mercury cadmium telluride detector), was employed for the analysis. The settings were: transmittance mode, a spectral range of 4000–1200 cm^−1^, 100-μm step size scanning (spatial resolution), 100–100 μm aperture, and 64 co-added scans at a spectral resolution of 4 cm^−^^1^ [7,13,20].

Microscopic counting was performed according to Corami et al. [7,20]. Microscopic counting has been employed for bacteria, phytoplankton, pollen, spores, and microplastics as well [21,22,23,24,25,26,27,28,29,30,31]. A significant advantage of microscopic counting is that there is no doubt about how many organisms, cells, or particles are present within reliable computable limits and degrees of chance. When filters are employed as a support for counting, the measurement of complete filters is very time-consuming [28,30,31]. However, analyzed filter areas, i.e., counting areas or count fields, need to represent the entire filter to avoid issues regarding representativeness and reproducibility. Since the loading of the filters cannot be known in advance, counting areas with different abundances should be considered to avoid issues regarding the accuracy of the extrapolation of microplastics, organisms, cells, or bacteria findings.

In our study, at least 14 known-sized areas (i.e., count fields) were randomly chosen with no overlapping on the surface of the filter (the different approaches to choosing representative measurement areas are in the Supplementary Information, Figure S1). Moreover, a significant number of particles (250–350 particles per count field) were analyzed using the PARTICLES WIZARD of the Omnic™ Picta™ software. The spectral background was acquired on a clean point in each count field. The IR spectrum was retrieved for each particle, and the spectral background was deduced; the resulting spectrum was then compared with several reference libraries (the list of reference libraries is in the Supplementary Information). In PARTICLE WIZARDS, particles were identified and counted when the identification match percentage was ≥65%; when operating with this software section, the optimal range of match percentage is between 65% and 75%. Moreover, particle sizes (length and width) were collected using the Imaging of PARTICLE WIZARDS.

The total number of SMPs and APFs per organism was then calculated according to Equation (1) (modified from Corami et al., 2020b [13]):

(1) $$\frac{N_{\mathrm{tot}}}{\mathrm{Specimen}}=\frac{\left(n*F\right)}{n\;\mathrm{specimens}}$$

where n = SMPs or APFs counted on every field, n specimens = the total number of organisms analyzed, and F = count factor, calculated as follows:

(2) $$F=\frac{\mathrm{Total}\;\mathrm{area}\;\mathrm{of}\;\mathrm{the}\;\mathrm{filter}}{\mathrm{Area}\;\mathrm{of}\;a\;\mathrm{count}\;\mathrm{field}\;*\;n\;\mathrm{count}\;\mathrm{fields}}$$

The weight of microplastics per specimen can be calculated according to Equation (3) (modified from Corami et al., 2020b [13]):

(3) $$\frac{W_{\mathrm{tot}}}{\mathrm{specimen}}=\frac{N_{\mathrm{tot}*V*\rho }}{n\;\mathrm{specimens}}$$

where W_tot_ = total weight of SMPs or APFs, n specimens = the total number of organisms analyzed, V is the volume of each particle calculated based on its AR, and ρ is the identified polymer’s density, additive, plasticizer, etc.. The aspect ratio (AR); [13,32,33] is the ratio between the maximum length (L) and the maximum width (W) of the smallest rectangle (bounding box) enclosing the particle chosen with the Imaging of PARTICLE WIZARDS, employed for the analysis. When the AR ≤ 1, particles are considered spherical; when the AR ≤ 2, particles are elongated/ellipsoidal. When the AR ≥ 3, particles are considered cylindrical. The volumes of SMPs and APFs can be calculated according to their geometrical shape (i.e., sphere, ellipse, and cylinder).

### 2.5. Statistical Analysis

The abundance and distribution of SMPs and APFs, as well as their weights, are expressed as the average number of particles per organism. Statistical analyses were performed using STATISTICA software (TIBCO, Palo Alto, CA, USA). Fisher’s exact test was performed to test whether the variances of the abundance of SMPs and APFs were homogenous (F test, α = 0.05). After invalidation of the homogeneity of variances, non-parametric statistical tests were performed to assess significant differences in the abundance of ingested APFs, SMPs, and other components of the microlitter. While the Kruskal–Wallis test (*p* < 0.05) was employed for multiples comparison, the Mann–Whitney U test (*p* < 0.05) was performed for pairwise comparisons. Since particles’ abundance data are count data, they follow a Poisson distribution [20,34,35]; Poisson’s confidence interval was calculated accordingly.

## 3. Results

### 3.1. SMPs Ingested by Blackfly Larvae

SMPs and APFs were not detected on reagent and procedural blanks. Contamination was minimized during all steps of the pretreatment and analysis.

**Figure 2:**
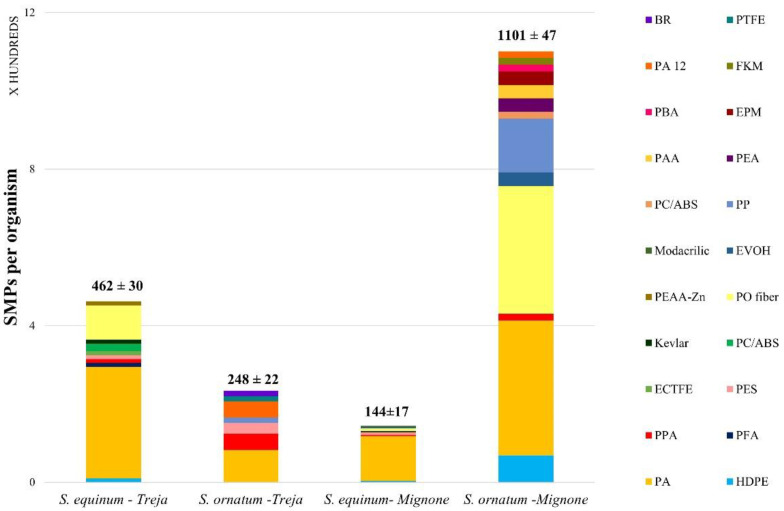
The average abundance of SMPs per organism in the two species of blackfly larvae under examination, *Simulium equinum* and *Simulium ornatum* (20 organisms per species for each sampling site were analyzed). The fiducial interval according to Poisson’s distribution is reported for each species in the sampling sites studied. The distribution of polymers ingested is shown as well. Complete names of the polymers can be found in Table 1.

**Figure 3:**
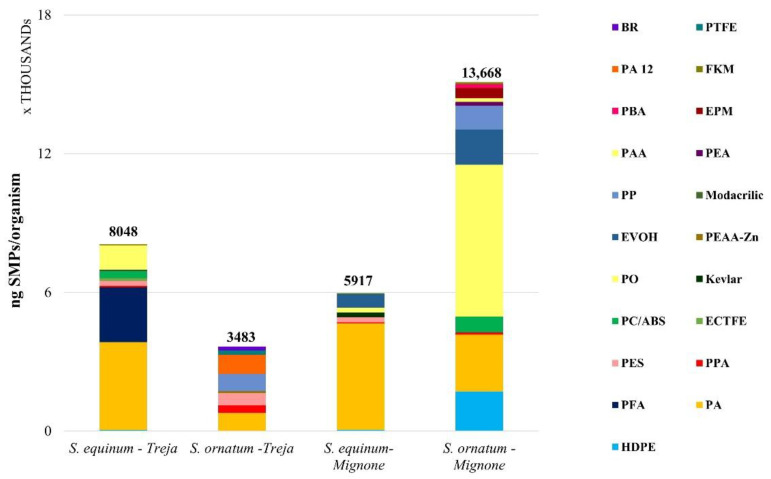
Weight of ingested SMPs (ng SMPs/organism) by *S. equinum* and *S. ornatum* collected in the Treja and Mignone rivers.

The complete list of polymers identified and quantified is reported in Table 1. The abundance of the SMPs ingested (n SMPs/organism) by the specimens of *S. equinum* and *S. ornatum* in the two rivers under study is shown in Figure 2, while the weight of the ingested SMPs is shown in Figure 3. The fiducial interval (FI, or confidence interval) was calculated according to Poisson’s distribution.

Polymers with a wide range of densities were identified and quantified, e.g., from PP (density = 0.9005 g cm^−3^) to PTFE (density = 2.2 g cm^−3^) and FKM (density = 2.1 g cm^−3^). The match percentage (i.e., the correlation coefficient between the measured spectrum and the reference spectrum for each polymer identified or the match %) was in the optimal range (65–75%) for all of the identified polymers. Moreover, the match percentage of several spectra identified in the analyzed samples was well above 75% of the optimal match percentage (i.e., >85%, HDPE, PO, PP, PTFE). Some spectra are shown as examples in the Supplementary Information (Figure S1). Only optimally identified SMPs (match % ≥ 65%) were quantified.

The highest abundance of SMPs was shown by the *S. ornatum* collected in the Mignone River (1101 ± 47 SMPs/organism) at almost five times higher than the abundance of the same species collected in the Treja River (248 ± 22 SMPs/organism). Regarding *S. equinum*, the specimens of the Mignone River showed the lowest abundance (144 ± 17 SMPs/organism) at almost 70% lower than the abundance of the same species in the Treja River (462 ± 30 SMPs/organism).

**Figure 4:**
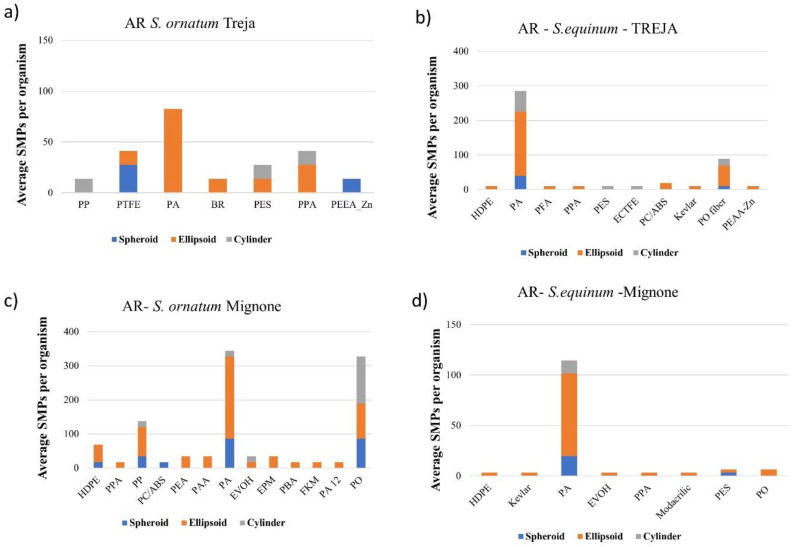
Aspect ratio (AR) of the polymers identified and quantified in specimens of *S. ornatum* (**a**,**c**) and *S. equinum* (**b**,**d**) under examination. The number of the spheroid, ellipsoid, and cylinder particle shapes is reported for the average abundance of each polymer identified and quantified via microscopic counting.

Most of the SMPs ingested by the two species in the two rivers studied were less than 52 μm in length. According to their AR (Figure 4), ellipsoidal particles were prevalent for all the polymers identified. The average length of particles in the Treja River, ingested by *S. equinum* (46 μm), was higher than that of the *S. ornatum* (39 μm); in contrast, the latter ingested larger particles in the Mignone River (52 μm and 42 μm for *S. equinum* and *S. ornatum*, respectively).

### 3.2. APFs and Other Components of Micro-Litter Ingested by Blackfly Larvae

The same pretreatment method allowed for simultaneous extraction of the SMPs and APFs, which were then filtered on the same filter. Afterward, APFs were quantified and detected concurrently with SMPs in the same analysis via MicroFTIR.

**Figure 5:**
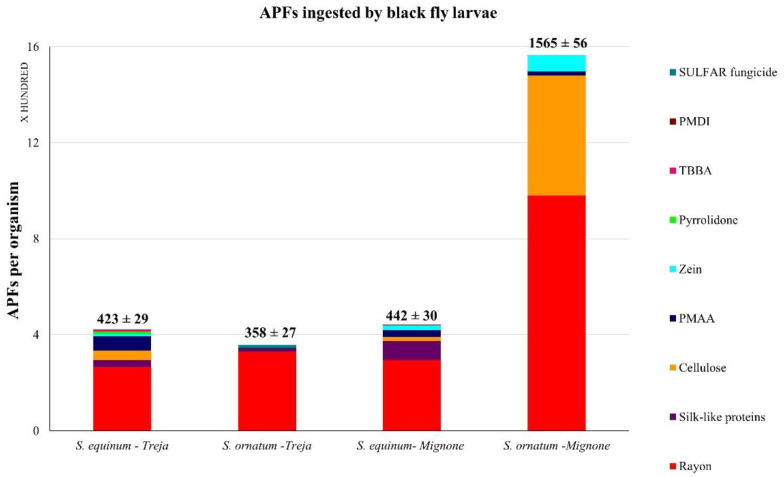
The average abundance of APFs per organism in the two species of blackfly larvae under exam, *Simulium equinum* and *Simulium ornatum* (20 organisms per species for each sampling site was analyzed). The distribution of ingested additives, plasticizers, and other microlitter components is also shown. Rayon is a non-plastic synthetic fiber, which is preeminent in all the specimens studied. Simuliidae can ingest larger particles if compressible; some rayon fragments in *S. ornatum* in the Mignone River were >150 μm in length. The fiducial interval according to Poisson’s distribution is reported for each species in the sampling sites studied.

**Figure 6:**
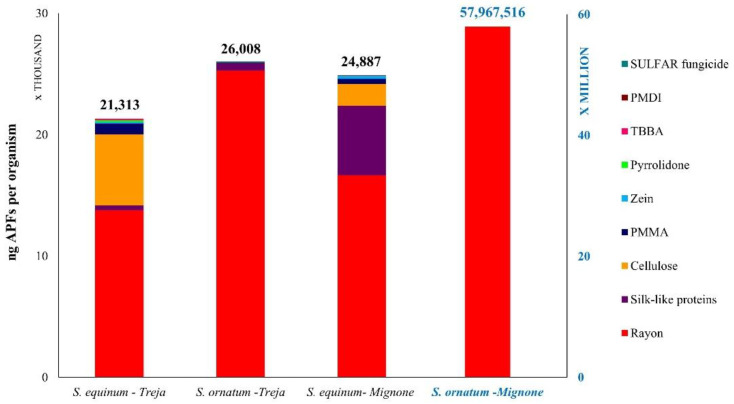
Weight of ingested APFs (ng APFs/organism) by *S. equinum* and *S. ornatum* collected in the Treja and Mignone rivers.

The abundance of the APFs ingested (n APFs/organism) by the two species investigated is shown in Figure 5. *S. ornatum* in the Mignone River showed the highest abundance of APFs (1565 ± 56 APFs/organism) at almost four times higher than the abundance of APFs in *S. equinuum* (442 ± 30 APFs/organism). The lowest abundance of APFs was observed in the Treja River, once again in *S. ornatum* (358 ± 27 APFs/organism), while *S. equinum* showed a comparable concentration (423 ± 29 APFs/organism) to that observed in the Mignone River. The weights of the AFPs ingested by *S. equinum* and *S. ornatum* in the two rivers are shown in Figure 6. The specimens showed approximately the same weight of APFs (ng/organism), except for *S. ornatum* in the Mignone River, which showed the highest weight of APFs (58 mg/organism). Rayon was the most represented among the APFs observed.

**Figure 7:**
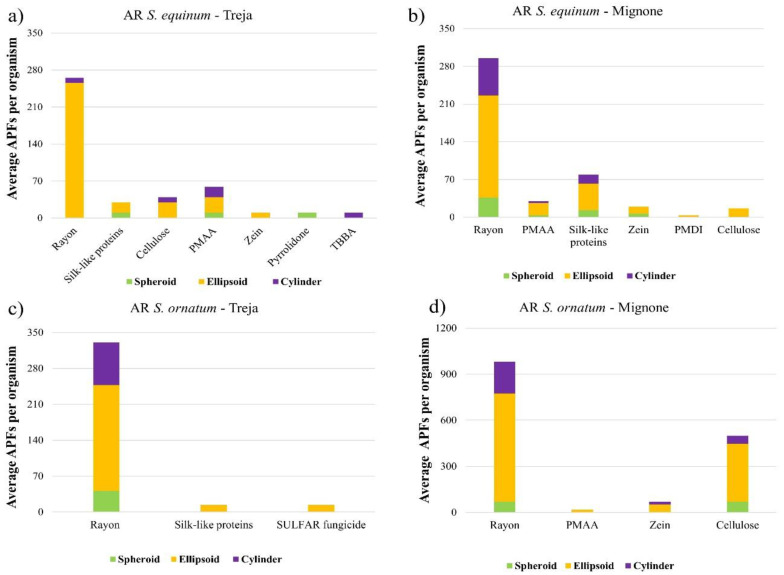
Aspect ratio (AR) of the APFs identified and quantified in specimens of *S. equinum* (**a**,**c**) and *S. ornatum* (**b**,**d**) under examination. The number of the spheroid, ellipsoid, and cylinder particle shapes is reported for the average abundance of each particle identified and quantified via microscopic counting.

As noted for the AR of SMPs, the ellipsoidal shape was prevalent for APFs (Figure 7). The average sizes of the APFs ingested by *S. ornatum* (length 70 μm, width 35 μm in the Mignone River; length 69 μm, width 32 μm in the Treja River) were higher than those ingested by *S. equinum* (length 55 μm, width 29 μm in the Mignone River; length 55 μm, width 28 μm in the Treja River). It should be noted that the high abundance and amount of rayon observed in the *S. ornatum* in the Mignone river is due to the presence of fragments higher than 150 μm in length.

## 4. Discussion

### 4.1. SMPs Ingested by Blackfly Larvae

The variances of polymer distributions for *S. equinum* and *S. ornatum* in the two rivers were different (F test, α = 0.05); according to the non-parametric Kruskall–Wallis test, the observed differences in the abundances and polymer distributions for both species in the two rivers were statistically significant (*p* < 0.05). Statistical analysis (Mann–Whitney U test, *p* < 0.05) showed that the differences in the SMPs’ observed abundances for the same species in the two rivers under study were significantly different, just as the SMPs’ abundances of the two species studied in the same river were also consistently dissimilar.

Specimens of *S. ornatum* showed a wider variety of polymers ingested than the organisms of *S. equinum* in the studied rivers. Several different factors (e.g., environmental, chemical, biological, etc.) could affect the ingestion of SMPs by blackfly larvae. The observed differences might be related to the type of polymer, the sources and pathways that the specific polymer followed before entering the riverine water, and where the blackfly larvae of the two species are located in these rivers.

The most abundant polymer was PA; this was followed by PO (maximum value for *S. ornatum* in the Mignone River, 327 ± 14 SMPs/organism, 2464 ng/organism), which has many usages in fabrics and textiles and may have diffuse sources. PA’s abundance in the Treja River was 285 ± 19 SMPs per organism (3806 ng/organism) of *S. equinum* and 83 ± 7 SMPs per organism (786 ng/organism) of *S. ornatum*, while in the Mignone River, PA’s abundances were 115 ± 14 SMPs/organism (4605 ng/organism) and 344 ± 15 SMPs/organism (2464 ng/organism), respectively. Another polymer present in all of the organisms in both rivers is PPA, primarily employed in electronics and electrical equipment. As assumed for PAA and EPM ingested by *S. ornatum* in the Mignone River, sources could also be diffuse for PPA. PES was ingested by the two species in the Treja River and only by *S. equinum* in the Mignone River. It should be noted that the shapes of the PES particles ingested by *S. equinum* were quite different, i.e., ellipsoidal and cylindric in the Treja River, while ellipsoidal and spheroidal in the Mignone River; this might support the notion that the pathways to the two rivers and the larval preference for the sizes of ingested particles may be somewhat different.

Regarding fluorinated polymers, the two species in the Treja River ingested a variety of them, i.e., PFA, ECTFE, and PTFE, while the *S. ornatum* in the Mignone River ingested only FKM. As a group of polymers, fluorinated polymers are employed for several purposes, from insulation to piping, waterborne coating systems, cookware, fabric and carpet protection, and the mechanical and automotive industries, to name a few. The presence and pathways of these polymers are a function of their widespread and extensive use; the ingestion by blackfly larvae may have been affected by the fragments’ shape.

The ingestion of BR in *S. ornatum* in the Treja River should be highlighted; 70% of this polymer is employed in the manufacturing of tires. Tire wear particles can enter the environment through atmospheric transport, WWTP effluents, and road runoff, and then accumulate in sediments and surface waters [36] where biota can ingest them. 

Some other studies have dealt with the presence of MPs by riverine insects [10,11,12,37,38,39,40,41,42,43]. Some of these have dealt with the ingestion of MPs by riverine insects [10,11,12,40,41,42,43]; however, the insects studied were not Simulidae, and some studies were mainly exposure experiments to few native polymers. Caddisfly cases (Trichoptera) from the same area of the Mignone River were investigated for the presence of plastics [39]. Nevertheless, the fragments studied had sizes (~1 mm) well above those observed for the ingested SMPs by the two species under examination here, and they were analyzed only by a visual exam (microscopical examination); thus, the polymers were not properly identified.

The polymers identified and quantified in this study were neither virgin nor native; they were discharged into the environment, and they reached the rivers through, e.g., atmospheric transport, rains, winds, and soil runoff. They were finally ingested by *S. equinum* and *S.ornatum* in the two rivers where specimens were collected.

Furthermore, a wide variety of polymers were identified and quantified thanks to the pretreatment method, which allowed for the recovery of low-density polymers, e.g., PE and PA, and high-density polymers, such as PTFE and FKM. It should be highlighted that the experimental conditions used for pretreatment did not affect particle size [19] and made it possible to identify PA and other polymers unambiguously [7,19], which allows for a more adequate and representative quantification of what is ingested by the organisms.

### 4.2. APFs Ingested by Blackfly Larvae

According to the Mann–Whitney U test, significant differences were observed for the same species in the same rivers in the two rivers studied and for the two species in the same river (*p* < 0.05). The variances of the APFs’ distributions for *S. equinum* and *S. ornatum* in the two rivers were different (F test, α = 0.05). According to the non-parametric Kruskall–Wallis test, the differences observed in abundance and distribution of APFs for both species in the two rivers were highly statistically significant (*p* < 0.01).

Non-plastic synthetic fibers, i.e., rayon, and natural fibers, such as cellulose, are often identified in several organisms [5,21,44,45,46,47]. Several lotic insects produce silk-like proteins or silk, e.g., caddisflies, aquatic moths, and dipterans [48,49]. Rayon and silk-like proteins were predominant in both species in the Treja and Mignone rivers. While the blackfly larvae produce silk-like proteins, it should be noted that washing machine discharges can contain rayon fragments, which are then released into the environment [50] after flowing through wastewater treatment plants. Another potential source of rayon in the environment is the decomposition of cigarette butts unwisely abandoned by tourists in the woods at the most visited places near the Treja and Mignone rivers.

However, additives and plasticizers are often overlooked. These compounds are added to polymers to impart specific features and can be released into the environments when plastic objects and macroplastics are broken into smaller fragments [51,52]; thus, they can be employed as proxies of the presence of polymers. Moreover, additives and plasticizers can exert toxic effects on biota [14]; therefore, the quantification and the identification of these compounds are relevant for an in-depth knowledge of plastic pollution and the potential hazards for biota in the whole trophic web.

Additives are, e.g., PMAA (polymethylacrylamide) employed as a flocculant in wastewater treatment and coatings such as those found in specimens for both rivers (i.e., PEAA-Zinc); TBBA (tetrabromobisphenol A), employed as a flame retardant and present in sewage sludge; and PMDI (methylene diphenyl diisocyanate), which is employed for polyurethane manufacturing.

Cellulose ingested by the organisms might not be human-made but rather part of the food they usually eat. The other compounds ingested by the organisms have the most diverse usages. While Sulfar^®^ is a fungicide used for vine cultivation, pyrrolidone is employed in pharmaceutics and as an additive for inkjet cartridges; these compounds are generally contained in plastic packaging, and their residues may have remained on plastic fragments that were subsequently ingested. Zein is a component of biopolymers.

Due to their sizes (<50 μm in length), most of these compounds may reach the two rivers alongside water leaving the treatment plants in the area (for instance, near the sampling site at the Treja River, there is a wastewater treatment plant at Mazzano Romano). It is worth noting that polyurethane was not found in the specimens collected, but the specific additive PMDI was identified. Hence, additives and plasticizers may be significant proxies of plastic polymers.

## 5. Conclusions

This study is the first to show that blackfly larvae (Simuliidae), members of a cosmopolitan insect family employed to test the quality of river waters via several status assessment methods, can ingest SMPs and APFs in their own habitat. Moreover, this is the first study to show that additives and plasticizers can be ingested by biota. The quantification and identification of additives and plasticizers will be relevant to assessing the MPs’ pollution and the potential threat they may pose to biota.

The pretreatment method allowed for retrieval of the ingested SMPs and APFs simultaneously and efficiently because the yield is >90%. Moreover, the pretreatment method employed did not further denaturate the polymers that could be optimally identified, as shown by the identification of PA; this polymer can be easily overlooked due to the temperatures and aggressive reagents employed, resulting in an underestimation of the actual MP abundance.

Statistically significant differences were observed intra-species in the abundance of SMPs and APFs at both the Treja and Mignone sites under examination, which are used to survey river water quality. Further, relevant statistical differences were observed inter-species in each river under investigation. Based on these preliminary results, it is somewhat difficult to address differences related to the feeding behavior of the larvae of these two species in the two rivers studied; these differences may be related to several environmental, ecological, biological, and chemical factors. However, the results of this study can be relevant to further thorough studies of the various links among the factors mentioned above. 

Investigating what has been ingested by the larvae of *S. ornatum* and *S. equinum* may account for the environmental impacts, hazards, and threats that pollutants such as SMPs and APFs may pose to biota and the good environmental quality status of river waters. Since Simulidae are commonly used in biomonitoring to assess riverine waters’ ecological conditions (European Water Framework Directive 2000/60/EC), these preliminary data could aid further in-depth investigations of blackfly larvae and their potential role as bioindicators of microplastic pollution.

## Figures and Tables

**Table 1:** List of the polymers identified and quantified in the specimens of *S. equinuum* and *S. ornatum*, collected in the Treja and Mignone rivers.

HDPE	High Density Polyethylene
PA	Nylon 6
PFA	Pefluoroalcoxy Fluorocarbon
PPA	Polyphtalamide
PES	Polyester
ECTFE	Ethylene chlorotrifluoroethylene
PC/ABS	Polycarbonate/Acrylonitrile Styrene Butadiene
ARAMID	Aramid
PO	Olefin fiber
PEAA-Zn	Polyethylene acrylic acid copolymer—Zinc salt
EVOH	Ethyl vinyl alcohol
MODACRILIC	Modacrilic
PP	Polypropylene
PEA	Polyethylacrylate
PAA	Polyarylamide
EPM	Ethylene propylene rubber
PBA	Polybutylacrylate
FKM	Fluoroelastomer
PA 12	Grilamid tr 55
PTFE	Polytetrafluoroethylene
BR	Butadien rubber

## Data Availability

Not applicable.

## Supplementary Materials

The following supporting information can be downloaded at: https://www.mdpi.com/article/10.3390/toxics10070383/s1, Figure S1: Polymer spectra collected, as an example, some of the spectra identified with match percentages greater than 85% are shown.
