# Supplementary material for: Occurrence and Characterization of Small Microplastics (<100 μm), Additives, and Plasticizers in Larvae of Simuliidae
Source: Toxics. 2022 Jul 10;10(7):383. doi: 10.3390/toxics10070383 (PMC9321584; doi:10.3390/toxics10070383)
Supplement: Supplementary file 1 [file toxics-10-00383-s001.zip › toxics-1813279-supplementary.pdf]

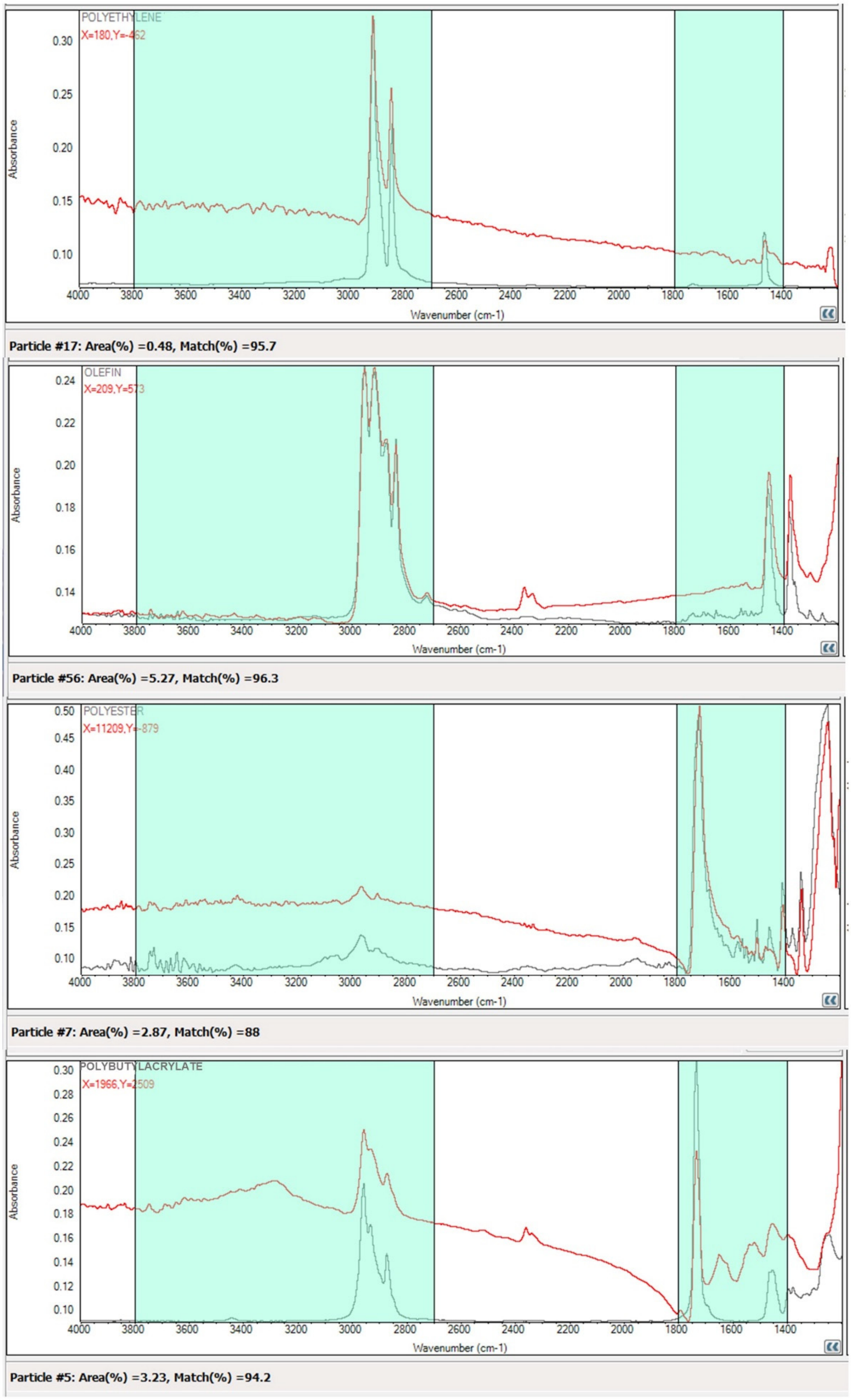

Figure S1 As an example, some of the spectra identified with match percentages greater than 85% are shown

List of reference libraries employed for the analysis via MicroFTIR, software PARTICLE WIZARDS, Omnic™ Picta™

Synthetic fibers by Microscope

Plastic Fibers

Polymer additives and plasticizers

HR Polymer additive and plasticizers

HR Hummel Polymer and plasticizers

HR Nicolet Sampler Library

HR Sprouse Polymer additives

HR rubber compounding materials
